# Sir R. Douglas Powell on Recent Advances in Practical Medicine

**Published:** 1899-08-05

**Authors:** 


### SIR R. DOUGLAS POWELL ON RECENT ADVANCES IN PRACTICAL MEDICINE.

In the Address in Medicine delivered last Wednesday at the annual meeting of the British Medical Association Sir Douglas Powell dealt with some of the most important problems with which physicians of the modern school are now concerned, and he dealt with them in a way which should demonstrate once for all, if that were necessary, that although there may be, and no doubt are, plenty of medical men who are easily swayed by fashion, and thus tempted to accept untried or half-tried theories, there are also men among the leaders of our profession who, without being second to any in the possession of the scientific imagination, are ready to maintain that it is scientific only when it is used to suggest new lines of research, not when it is so employed as to become a mere parent of hypotheses.

### "The Third Circumstance."

The strong point brought out by Sir Douglas Powell's address is the importance of what he calls "the third circumstance," among those which are essental to the production of a disease by a micro-organism, that namely which renders the specific microbe virulent to its host. "It has long been clear," he says, "to every observant physician, who has on the one hand even superficially kept in view the results of bacteriological inquiry, and who has thought of the incidences of such infective diseases as he happens to meet, that we carry about with us in our accessible mucous tracts, and especially in our naso-oral and respiratory passages, amidst other unconsidered trifles," samples of the organisms specific to many diseases. We are tenanted by these varied organisms from time to time, singly or in small colonies, and they remain inert only through want of opportunity. Bacteria surround us on all sides, and from time to time obtain a temporary but abortive lodgment within us. Virulent catarrh, diphtheria, pneumonia, influenza, tuberculosis, erysipelas, perhaps rheumatism and probably in epidemic times some of the other infective diseases, would be represented amongst our domesticated or casual occupants.

To acquire the disease, however, it is not enough merely to have the poison germ; there are other intermediate or antecedent circumstance of dosage, and acquired or inherited susceptibility—that subtle malformation of tissue in certain organs which renders them weak in resistance to certain forms of attack. Let a period of depression come over us, involving some slight change in our blood or our tissues, and one or other of these organisms may find the opportunity for aggressive cultivation.

### A Common Cold.

We virtually know that this is so in the case of a common cold. Acquired by a momentary chill at an open door, or through wet boots, such a catarrh becomes at once a highly contagious disease, and will "run through the house." There can be no doubt that the catarrh is associated with the cultivation of an organism, which equally probably had pre-existed in some part of the nasal surface, but only became virulent under the conditions set up by chill. There are many people who are constantly catching colds, which are doubtless due to successive crops of an ever-present organism. But the organism we cannot reach; "we might as well do battle with a sunbeam." Our treatment is to fortify the host.

### Other Examples.

Sir Douglas Powell emphasises this point in regard to the importance of the "third circumstance" by the following case: "Some few years ago I saw in consultation a medical friend who had pneumonia of a pythogenic type; but in response to my inquiry about drains I was informed that he had been in the house for some years, that he had a healthy family of young children, and that he himself had been in perfect health until he went out thinly clad on a cold night and got definitely chilled. He died after the lapse of a day or two, and on investigation a free communication was found to exist between the main drain and his surgery. This gentleman must for some months have been occupied by microbes which did not become operative under the control of a robust vitality until that vitality was temporarily impaired."

Nor does this hold good merely in regard to the more commonly recognised infections. Rheumatism, a disease which runs in families although an infection, is a case in point. It is hardly possible to conceive that chorea is not a manifestation of the rheumatic poison, which is most probably a micro-organism. A mental shock is the usual intervening etiological factor in chorea, which presumably weakens the resistance of the nerve centres upon which the toxic influence falls. It is hardly possible that the mental shock and infection should have coincided in time.

### Serum Therapeutics.

The subject, however, takes on another aspect when we consider the influence of the various microbic factors engaged in the production of an illness, and this brings us to consider the question of serum therapeutics. It is an immense achievement if we have acquired the knowledge that every infection requires a separately-prepared serum for its treatment. It gives promise of adding to our successes, but in the meantime it explains many of our failures. It is useless to employ an antistreptococcus serum for a pneumococcus infection, and even the two organisms, streptococcus and staphylococcus, which seem to work most cordially in couples, require a separate treatment.

In pneumonia it is remarkable that in every variety of the disease the characteristic pneumococcus is invariably to be found, and this coccus may be the microorganism most conspicuously present in those secondary lesions with which pneumonia is often complicated, and which are often attributed to it, such as empyema, infective endocarditis, &c. Yet there are good reasons to doubt whether the pneumococcus organism alone, unassisted by some of its pyogenic *confrères*, is ever able to bring about those secondary lesions which are usually attributed to it. Clearly the presence of another microorganism may be the "third circumstance" which makes the development of the disease possible, and unless the nature of this other organism can be discovered serum therapy directed against the pneumococcus may be of but small avail.

Tuberculosis.

Of still further importance is the "third circumstance" in regard to the etiology of tuberculosis. After insisting on the gradual but very considerable reduction in the mortality from phthisis which has taken place during the last half century in response to measures which have had no direct reference to any special bacillus element in the disease, but have chiefly had to do with amending the conditions which are now often spoken of as "predisposing causes," all of which come under the head of the "third circumstance," Sir Douglas Powell goes on to say: "The question of the prevention of consumption, which should have been calmly and forcefully considered as heretofore by experts in human and veterinary medicine, has become the subject of popular agitation with all the reckless exaggeration and no little threatening of the social tyranny which such agitations tend to produce. . . . . That it is *au fond* a right grouping to place all the previously ascertained causes of consumption amongst the predispositions, and to regard the disease itself as produced by definite infection or contagion through the inhalation of sputum dust, or by the ingestion of tuberculous foods, has been accepted at least for the last ten years; but we must not discontinue to attach a due importance to the other etiological factors because one of them may seem *primâ facie* to be sufficient. To set heredity at nought, to regard climatic considerations as of no importance, and to state that the disease is always acquired by direct contagion or infection is, in my opinion, to ignore much that is true, and to magnify that which should be carefully guarded from exaggeration." Finally, he insists that although we may hope, by careful sanitation, by destroying and diminishing by the careless distribution of bacillary dust, and withdrawing from human consumption tuberculous foods, to still further diminish the mortality from consumption, we cannot afford, on the other hand, to withdraw or relax precautions dictated by observations which, however much they may be amended, are sound in themselves. We must not too much concentrate our attention on the bacillus, for the circumstances under which it can flourish are of equal importance. General sanitation and cleanliness form our first line of defence against all these organisms, and not against that of tubercle alone, and wherever conditions of insanitation, dampness, deficient sunlight, and the prevalence of favouring diseases are present there aggressive activity in micro-organisms may again be looked for.

